# Supplementary material for: Molecular Characterization and Functional Analysis of Odorant-Binding Proteins in Ectropis grisescens
Source: Int J Mol Sci. 2025 May 10;26(10):4568. doi: 10.3390/ijms26104568 (PMC12110804; doi:10.3390/ijms26104568)
Supplement: Supplementary file 1 [file ijms-26-04568-s001.zip › ijms-3592162-supplementary.pdf]

Supplementary Materials

Table S1. The amino acid identity among the 18 *E. grisescens* OBPs

	EgriGOBP1	EgriGOBP2	EgriPBP1	EgriPBP2	EgriPBP3	EgriOBP1	EgriOBP2	EgriOBP3	EgriOBP4	EgriOBP5	EgriOBP6	EgriOBP7	EgriOBP8	EgriOBP9	EgriOBP10	EgriOBP11	EgriOBP12	EgriOBP13
EgriGOBP1	100																	
EgriGOBP2	55.56	100																
EgriPBP1	46.00	45.07	100															
EgriPBP2	52.17	42.40	53.96	100														
EgriPBP3	47.06	45.41	52.71	62.05	100													
EgriOBP1	31.69	23.11	27.96	16.32	21.76	100												
EgriOBP2	0.58	5.79	15.33	25.31	9.90	20.45	100											
EgriOBP3	26.53	27.01	31.86	20.63	3.22	37.97	24.51	100										
EgriOBP4	33.89	28.77	33.82	34.25	27.36	34.57	26.92	44.30	100									
EgriOBP5	32.07	25.93	29.81	29.85	36.13	27.96	20.17	36.59	40.49	100								
EgriOBP6	34.62	25.59	33.01	34.04	31.68	25.65	25.57	38.27	30.69	60.39	100							
EgriOBP7	31.61	21.36	30.48	30.81	23.56	28.33	17.67	37.04	40.76	54.49	49.37	100						
EgriOBP8	33.85	25.64	29.72	29.56	26.24	33.33	22.97	39.88	39.16	30.43	35.91	34.66	100					
EgriOBP9	34.95	33.17	29.07	36.51	33.97	29.21	22.22	32.42	31.87	47.67	45.35	53.53	35.39	100				
EgriOBP10	34.67	31.1	31.65	35.12	33.17	22.64	35.90	28.43	31.44	32.78	31.41	28.28	24.77	34.65	100			
EgriOBP11	26.84	26.04	27.13	32.00	24.79	26.05	4.73	26.05	25.35	32.55	33.5	33.84	18.08	35.47	18.60	100		
EgriOBP12	13.79	15.06	24.09	2.91	24.73	21.51	19.66	29.65	26.03	23.64	17.14	25.30	27.20	10.87	18.37	28.13	100	
EgriOBP13	14.73	1.78	17.28	22.10	9.07	9.28	16.67	12.21	13.44	14.20	14.59	17.93	15.24	16.71	13.99	15.86	5.82	100

Table S2. The accession numbers of genes used for phylogenetic tree Construction.

OBPs Name	Accession#	OBPs Name	Accession#	OBPs Name	Accession#
BmorGOBP1	CAA64444.1	LbotOBP46	AXF48743.1	SlitOBP1	AKI87962.1
BmorGOBP2	CAA64445.1	LbotOBP47	AXF48744.1	SlitOBP10	ALD65884.1
BmorOBP1	NP_001140185.1	LbotOBP48	AXF48745.1	SlitOBP11	ALD65885.1
BmorOBP4	NP_001140188.1	MsepOBP1	JAV45912.1	SlitOBP3	AKI87964.1

BmorOBP6	NP_001140190.1	MsepOBP10	JAV45903.1	SlitOBP4	AKI87965.1
CsupPBP1	ADK66921.1	MsepOBP11	JAV45902.1	SlitOBP5	AKI87966.1
CsupPBP2	ACJ07123.1	MsepOBP2	JAV45911.1	SlitOBP6	AKI87967.1
CsupPBP3	ADL09140.1	MsepOBP5	JAV45908.1	SlitOBP7	AKI87968.1
EoblGOBP1	ACN29680.1	MsepOBP6	JAV45907.1	SlitOBP8	AKI87969.1
EoblGOBP2	ACN29681.1	MsepOBP7	JAV45906.1	SlitOBP9	ALD65883.1
EoblPBP1	ALS03847.1	MsepOBP8	JAV45905.1	SlitPBP1	AAY21255.1
EoblPBP2	ALS03848.1	MsepOBP9	JAV45904.1	SlitPBP2	AAZ22339.1
EoblPBP3	ALS03849.1	MvitPBP1	ANA06562.1	SlitPBP3	AIS72934.1
EoblPBP4	ALS03850.1	MvitPBP2	ANA06563.1	EgriGOBP1	ON380526
HarmGOBP1	XP_021192665.1	MvitPBP3	ANA06564.1	EgriGOBP2	ON380510
HarmGOBP2	XP_021192653.1	OfurOBP1	BAV56788.1	EgriOBP1	ON380521
HarmOBP16	AFI57165.1	OfurOBP10	BAV56797.1	EgriOBP2	ON380524
HarmOBP17	AFI57166.1	OfurOBP2	BAV56789.1	EgriOBP3	ON380515
HarmOBP18	AFI57167.1	OfurOBP3	BAV56790.1	EgriOBP4	ON380517
HarmOBP19	AFM93773.1	OfurOBP4	BAV56791.1	EgriOBP5	ON380527
HarmOBP31	ASA40067.1	OfurOBP5	BAV56792.1	EgriOBP6	ON380523
HarmOBP35	ASA40068.1	OfurOBP6	BAV56793.1	EgriOBP7	ON380525
HarmOBP36	ASA40069.1	OfurOBP7	BAV56794.1	EgriOBP8	ON380513
HarmOBP7	AEB54591.1	OfurOBP8	BAV56795.1	EgriOBP9	ON380518
HarmOBP9	AEB54592.1	OfurOBP9	BAV56796.1	EgriOBP10	ON380511
LbotOBP33	AXF48730.1	SexcPBP1	AXF80669.1	EgriOBP11	ON380516
LbotOBP39	AXF48736.1	SexcPBP2	AXF80670.1	EgriOBP12	ON380519
LbotOBP40	AXF48737.1	SfruPBP1	QKX94922.1	EgriOBP13	ON380522
LbotOBP41	AXF48738.1	SfruPBP2	QKX94923.1	EgriPBP1	ON380520
LbotOBP43	AXF48740.1	SfruPBP3	QKX94924.1	EgriPBP2	ON380512
LbotOBP44	AXF48741.1	SlitGOBP1	ABM54823.1	EgriPBP3	ON380514
LbotOBP45	AXF48742.1	SlitGOBP2	ABM54824.1		

Table S3. Primers used in this study

Genes	Forward primes (5'-3')	Reverse primes (5'-3')
**For gene cloning**		
EgriGOBP1	GTACGATGACATCACGGACGACTG	ACAGCTTCAATCATGGTG
EgriGOBP2	GAGCCAGGACGTGATGGAGGAG	GAGTCGGTGAGCAGGTT
EgriPBP1	TCGTGTTCGTGGTTGTGGTTGG	GTCGCTGATTAGGCCGTC
EgriPBP2	GCAGTCGTCTCAGGATGTGATGAAG	TTTAGTTACGGCGTCTGG
EgriPBP3	TGATCCACTGCCTCTCCACCAAG	GTGGCTCATGGCGAACTC
EgriOBP1	TCGGCTTGTAAGAACTGTGTGGTG	GGTCATCCGTCAGCTCTC
EgriOBP2	AGTCCCTGGTCCACCGCATATC	GCTGCTTGCAGTCATCAT
EgriOBP3	GGACGAAGACATGGCAGAACTAGC	TGCGTTTACTTTCTCCACC
EgriOBP4	CTGGAGGTTGCGATGCGATGAC	CTCCTACTTTCCCCAAGC
EgriOBP5	GCTGCCGGAGTAACTGACGAAG	TCGGACGGTATGCCAAAC
EgriOBP6	GCAGCGATCTCGGAAGAAGACAAG	CTCCGTGTTCTTTGCCGC
EgriOBP7	TGAAGACGCAGAGGAACTCAAGATG	AAGCCAAACTGTGACGC
EgriOBP8	GGGGTCTCCGAGGAGGATATAACG	CGGCCAGACTCGATTCTT
EgriOBP9	ACAGCAAAAGCAGCAACAAAGAAGG	TCACTGGTTTCGGCATCG
EgriOBP10	AATGCCTCCAATGAAGCCTCCATC	CGTCGTCAGAAGCCAGTC
EgriOBP11	CAGGACCGTGCCAATGGACAAC	GTTCCCGCAGTATTCGCC
EgriOBP12	CAACCTTCTCGCTACTGCCAACC	CGACATAGCCACATCCAT
EgriOBP13	AGAAACGATTGGGACCGAAATGACC	CTCCATAACTACCAGCGT
GAPDH	CTGCCTCCTACGATGCCATCAAAC	GGACGAGTGCGAGTCAC
**For prokaryotic expression**		
EgriGOBP2	CCC<u>AAGCTT</u>ATGAAGGACGTCACACTTGGGTTC	CC<u>CTCGAG</u>GGCTACTTG

Note: Restriction sites are underlined.
